# Evolution, Expression, and Function of Gonadal Somatic Cell-Derived Factor

**DOI:** 10.3389/fcell.2021.684352

**Published:** 2021-07-07

**Authors:** Chen-wei Hsu, Bon-chu Chung

**Affiliations:** Institute of Molecular Biology, Academia Sinica, Taipei, Taiwan

**Keywords:** gonad, ovary, oocyte, development, evolution, teleost, testis, *gsdf*

## Abstract

Fish gonads develop in very diverse ways different from mammalian gonads. This diversity is contributed by species-specific factors. Gonadal somatic cell-derived factor (Gsdf) is one such factor. The *gsdf* gene exists mostly in teleosts and is absent in many tetrapods, probably as a result of two gene losses during evolution. The *gsdf* transcript is expressed mainly in gonadal somatic cells, including Sertoli cell in testis and granulosa cells in ovary; however, these gonadal somatic cells can surround many types of germ cells at different developmental stages depending on the fish species. The function of *gsdf* is also variable. It is involved in germ cell proliferation, testicular formation, ovarian development and even male sex determination. Here, we summarize the common and diverse expression, regulation and functions of *gsdf* among different fish species with aspect of evolution.

## Introduction

The development of gonads is characterized by its diversity among species, while the development of other organs in different species follows similar rules. The best studied mechanism of gonad development is dictated by the XX/XY sex-determination system in many mammals. In humans and mice, gonadal sex is determined by *SRY* located on the Y chromosome. In males, SRY triggers the expression of downstream male factors including DMRT1 and SOX9 that control testicular development. In females, the absence of *SRY* facilitates the expression of female factors including FOXL2 and WNT4, which further trigger ovarian development (Bashamboo and McElreavey, 2016; She and Yang, 2017).

Fishes exhibit very diverse mechanisms of gonadal development, which can be common or distinct from other vertebrates. Fishes such as Nile tilapia (Li et al., 2015) and medaka (Matsuda et al., 2002) can exist as a single sex at a time (gonochoristic), while other fishes like black porgy (Wu et al., 2021) and clownfish (Fricke and Fricke, 1977) exist as hermaphrodites. The factors controlling fish sex can be genetic factors (Matsuda et al., 2002) or environmental factors, such as temperature for European sea bass (Koumoundouros et al., 2002) and social activity for anemone fish (Fricke and Fricke, 1977). To achieve this diversity, fishes preserve unique genes related to gonadal development during evolution.

**FIGURE 1:**
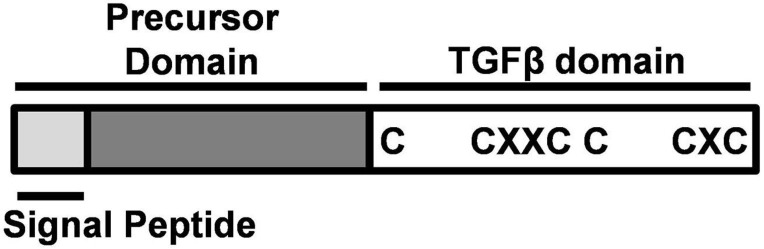
Structure of Gonadal somatic cell-derived factor (Gsdf). Gsdf is composed of a signal peptide and a precursor domain, which are cleaved after protein processing. The mature TGFβ domain is characterized by a cystine knot with three disulfide bonds. C, cystine; X, any amino acid.

**FIGURE 2:**
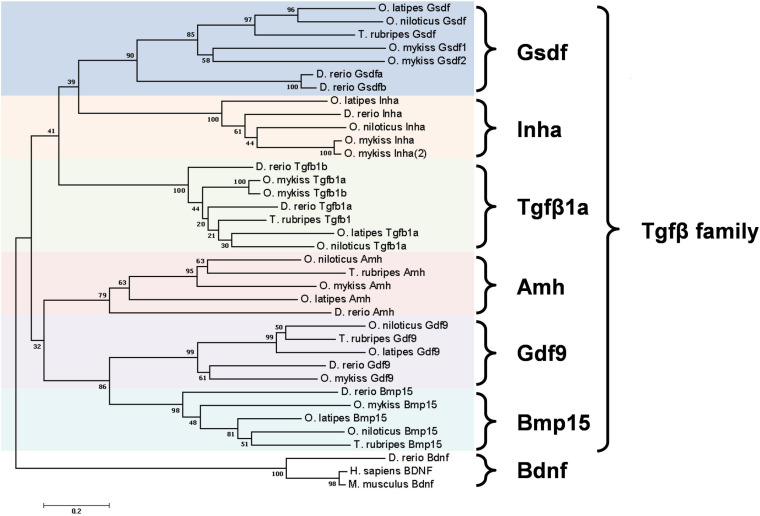
Phylogenetic tree of six Tgfβ family members in five teleost species. *Danio rerio* (zebrafish), *Oryzias latipes* (medaka), *Orechromis niloticus* (Nile tilapia), *Oncorhynchus mykiss* (rainbow trout), and *Takifugu rubripes* (Fugu). Phylogenetic analysis was performed by MEGA7 using nearest neighbor-joining method with 1000 bootstrap replicates. The unit of scale bar refers to the number of amino acid substitutions per site. Bdnf proteins were used as the outgroup. Amino acid sequence extracted from GenBank or Ensembl database are: *D. rerio* Gsdfa, ENSDARP00000149345; Gsdfb, ENSDARP00000134025; *O. latipes* Gsdf, ENSORLP00000042059; *O. niloticus* Gsdf, ENSONIP00000009618; *O. mykiss* Gsdf1, ENSOMYP00000032402; Gsdf2, ENSOMYP00000004997; *T. rubripes* Gsdf, ENSTRUP00000036138; *D. rerio* Inha, ENSDARP00000057347; *O. latipes* Inha, ENSORLP00000002713; *O. niloticus* Inha, ENSONIP00000016791; *O. mykiss* Inha, ENSOMYP00000007000; Inha(2), ENSOMYP00000010980; *D. rerio* Tgfb1a, ENSDARP00000060838; Tgfb1b, ENSDARP00000122056; *O. latipes* Tgfb1a, ENSORLP00000001563; *O. niloticus* Tgfb1a, ENSONIP00000007415; *O. mykiss* Tgfb1a, ENSOMYP00000099035; Tgfb1b, ENSOMYP00000101842; *T. rubripes* Tgfb1, ENSTRUP00000040849; *D. rerio* Amh, ENSDARP00000015395; *O. niloticus* Amh, ENSONIP00000006018; *O. mykiss* Amh, ENSOMYP00000058709; *O. latipes* Amh, ENSORLP00000006358; *T. rubripes* Amh ENSTRUP00000051375; *D. rerio* Gdf9, ENSDARP00000008475; *O. latipes* Gdf9, ENSORLP00000042760; *O. niloticus* Gdf9, ENSONIP00000016237; *O. mykiss* Gdf9, ENSOMYP00000052293; *T. rubripes* Gdf9, ENSTRUP00000050821; *D. rerio* Bmp15, ENSDARP00000054590; *O. latipes* Bmp15, ENSORLP00000010817; *O. niloticus* Bmp15, ENSONIP00000042526; *O. mykiss* Bmp15, XP_021470286; *T. rubripes* Bmp15, ENSTRUP00000048048; *D. rerio* Bdnf, ENSDARP00000069872; *O. latipes* Bdnf, ENSORLP00000031660; *O. niloticus* Bdnf, ENSONIP00000025919; *O. mykiss* Bdnf, ENSOMYP00000079299; *T. rubripes* Bdnf, ENSTRUP00000044236; *M. musculus* Bdnf, NP_031566.4; *H. sapiens* BDNF, NP-001137277.1.

A unique factor that controls gonad development in fish is gonadal somatic cell-derived factor (Gsdf). Gsdf is a secretory protein in the transforming growth factor β (TGFβ) family expressed mainly in teleost gonads. It is composed of a mature TGFβ domain and a precursor domain, which contains a signal peptide (Figure 1). The precursor domain is cleaved upon secretion, giving rise to mature Gsdf with the TGFβ domain. The TGFβ domain of Gsdf contains a characteristic cystine knot, but it lacks a glycine residue between 2nd and 3rd cystine. This signature is different from many other TGFβ proteins including Amh, Gdf9, and Inha (Vitt et al., 2001; Shibata et al., 2010). Phylogenetic tree further shows that Gsdf forms a unique clade different from other TGFβ proteins also known to be important for gonadal development (Figure 2).

Gonadal somatic cell-derived factor has attracted attention because it exists only in limited species to promote gonad development. In this review, we have compared the expression, regulation and functions of Gsdf in different species, and have delineated that Gsdf controls gonadal development via very diverse pathways.

## The Evolution of *Gsdf*

**FIGURE 3:**
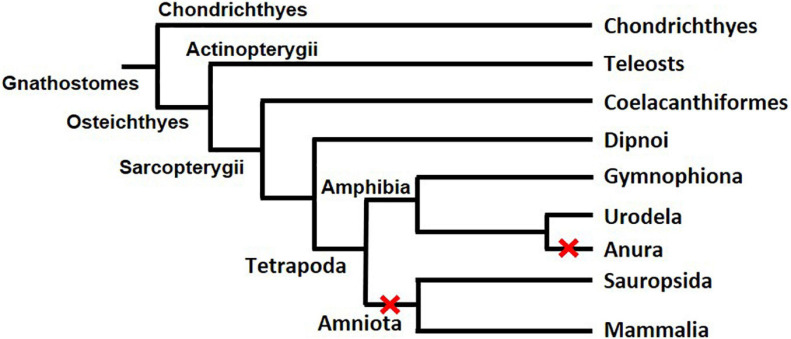
The evolution of *gsdf* in Gnathostomes. The two red X signs indicate two events of *gsdf* losses during the evolution of Tetrapoda. The figure is taken from a published article with permission (Biscotti et al., 2020).

First found in rainbow trout (*Oncorhynchus mykiss*) (Sawatari et al., 2007), *gsdf* exists mainly in teleosts (a branch of Osteichthyes) (Figure 3). BLAST and transcriptome analysis have further identified *gsdf* in non-teleost jawed fishes such as *Latimeria menadoensis* (Coelacanthiformes), *Protopterus annectens* (Dipnoi), and *Callorhinchus mili* (Chondrichthyes) (Forconi et al., 2013; Biscotti et al., 2018). However, whether *gsdf* exists in jawless fishes (Agnatha), the remaining class of extant fish, is still unclear. In addition, it is present in such tetrapods as *Cynops orientalis* (Urodela) and *Microcaecilia unicolor* (Gymnophiona), but absent in other tetrapods such as *Xenopus* (Anura), mammals and reptiles (Amniota). Phylogenetic analysis of various species that do or do not contain *gsdf* leads to the hypothesis that there are at least two losses of *gsdf* during the evolution of Tetrapoda (Figure 3) (Biscotti et al., 2020). One event of *gsdf* loss is in Anura, the other is in Amniota. The evolution of *gsdf* in ancient genome is an interesting question worth further investigation.

The synteny analysis comparing zebrafish (*Danio rerio*), human, spotted gar (*Lepisosteus oculatus*), and chicken (*Gallus gallus*) reveals that zebrafish *gsdf* locus contains conserved syntenies similar to that of human chromosome 4 (Gautier et al., 2011a; Yan et al., 2017). However, *gsdf* is within a breakpoint during chromosome rearrangement while other genes within the same syntenic locus are preserved. Synteny analysis comparing zebrafish, human, and spotted gar further indicates that *bmp15*, *gdf9*, and *gsdf* might originally be paralogs. The importance of *gsdf*, *bmp15*, and *gdf9* in ovarian development suggests that they might retain certain subfunctions of the ancestor gene (Force et al., 1999; Yan et al., 2017; Dalbies-Tran et al., 2020). Moreover, most genes within the syntenies are predominantly expressed in previtellogenic oocytes of *Oryzias latipes* and ovary of zebrafish (Gautier et al., 2011a). Thus, despite its male-dominant expression, *gsdf* is located within a conserved synteny inside a cluster of ovarian genes. Further analysis comparing synteny from two Tetrapoda clades that retain or lose *gsdf* may reveal the process of *gsdf* gene loss during evolution.

## The Expression of *Gsdf*

The *gsdf* gene is expressed in gonads, but the exact location of the gonad and timing of expression diverge among different species (Table 1). In most teleosts, *gsdf* is mainly expressed in gonadal somatic cells, and there are often more *gsdf* transcripts in testis than in ovary, suggesting the main role of Gsdf in testicular development. In the testis, *gsdf* is expressed in the Sertoli cells surrounding spermatogonia in all species studied to date (Sawatari et al., 2007; Shibata et al., 2010; Gautier et al., 2011b; Myosho et al., 2012; Kaneko et al., 2015; Jiang et al., 2019). Thus, Gsdf may support spermatogonial functions including their self-renewal, proliferation, and differentiation. In *Monopterus albus*, *Salmo salar*, and *Cynoglossus semilaevis*, *gsdf* expression is enriched in immature testis and decreased in mature testis (Zhu et al., 2016; Zhu et al., 2018; Kleppe et al., 2020), suggesting that *gsdf* functions in testis maturation during development.

**TABLE 1:** Expression of *gsdf* in gonochoristic fishes.

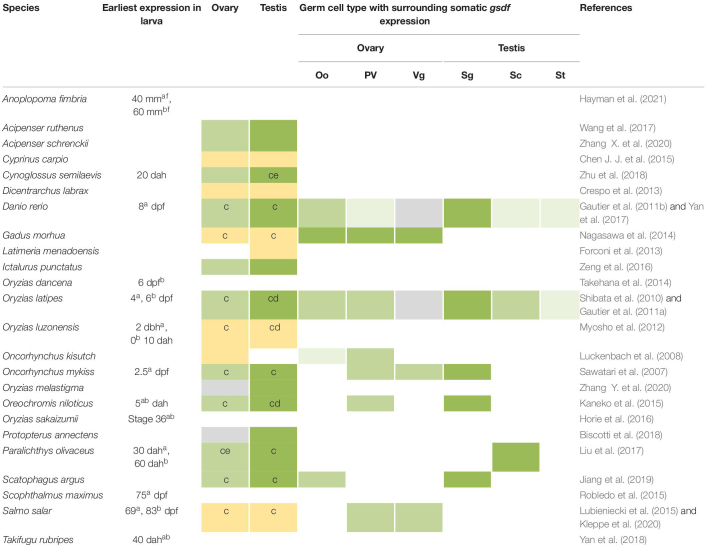

*^*a*^Start to be expressed. ^*b*^Higher expression in juvenile XY gonad. ^*c*^Expression in granulosa cell (ovary) or Sertoli cell (testis). ^*d*^Expression in sperm duct or efferent duct. ^*e*^Expression in germ cell (oocyte or spermatogenic cell). ^*f*^Developmental stage measured by mm fork length. dah, days after hatching; dbh, days before hatching; dpf, days post fertilization; mm, millimeter fork length; Oo, oogonia; PV, pre-vitellogenin; Vg, vitellogenin; Sg, spermatogonia; Sc, spermatocyte; St, spermatid. Color in the each box indicates the expression level. White, data is not available. Gray, no expression. Lighter green to darker green, lower expression to higher expression. Yellow, expression level is not specified in references.*

**FIGURE 4:**
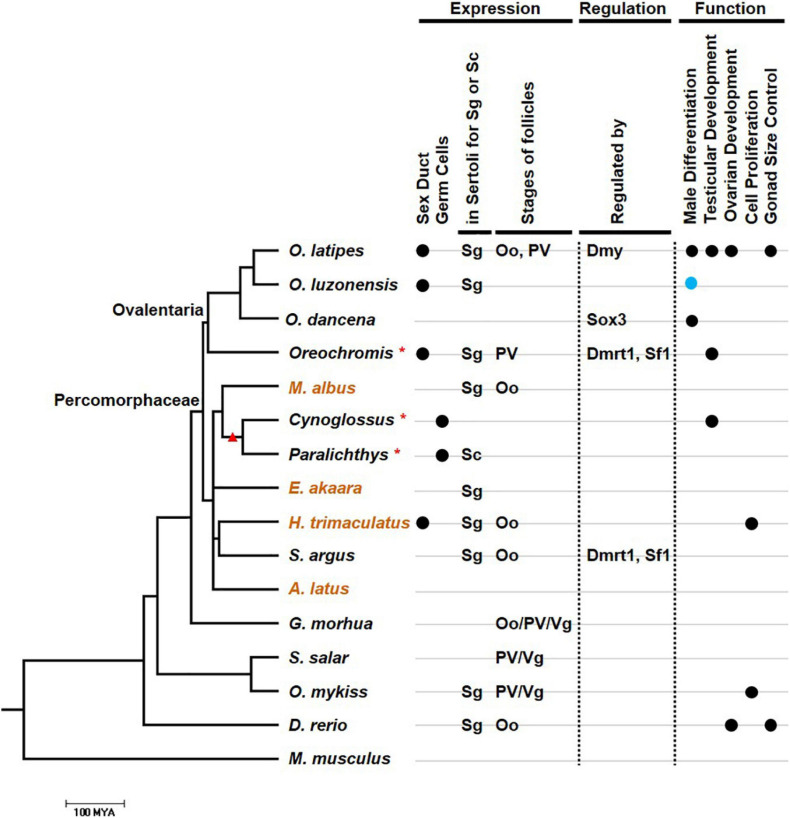
The relationship between the expression, regulation, function, and evolution of *gsdf*. The phylogenetic tree was drawn using information from the TimeTree database (http://www.timetree.org/resources). Species in the tree including those with *gsdf* expression in either Sertoli cell in testis or granulose cell in ovary. Mus musculus which does not have *gsdf* is an outgroup. Scale bar refers to 100 million years ago (MYA). When the evolutionary data of a particular species is missing in the TimeTree database, its genus is used for the compilation of the phylogenic tree and marked with an asterisk. These species include *O. niloticus* (Oreochromis), *Cynoglossus semilaevis* (Cynoglossus), and *Paralichthys olivaceus* (Paralichthys). Orange species names indicate hermaphroditic fish. The branch with a red triangle indicates Pleuronectiformes. The condition of additional expression of *gsdf*, including expression in efferent duct and germ cells, is labeled with black dots at the right part of first and second lane, respectively. The types of germ cells with predominant surrounding *gsdf* expression are listed in third and fourth lane. Oo, oogonia; PV, pre-vitellogenic oocyte; Sc, spermatocyte; Sg, spermatogonia; Vg, vitellogenic oocytes. The regulators are list in fifth lane while the functions are listed in lanes 6–9. The function of sex determination is labeled with a blue dot.

In *Halichoeres trimaculatus* and such Ovalentaria as *O. latipes*, *Oryzias luzonensis*, and *Oreochromis niloticus*, in addition to expression in Sertoli cells, *gsdf* is also expressed in epithelial cells of the intratesticular efferent duct, where no germ cells reside (Figure 4) (Shibata et al., 2010; Myosho et al., 2012; Horiguchi et al., 2013; Kaneko et al., 2015). For the female counterpart, no studies indicate expression of *gsdf* in ovarian cavity. The expression in ductal cells suggests that *gsdf* in these species may acquire additional function in the differentiation of male structure aside from gamete development.

In addition to somatic cells, *gsdf* is also expressed in germ cells of some fish species. In *Paralichthys olivaceus*, *gsdf* is additionally expressed in the cytoplasm of oocytes (Liu et al., 2017). This expression pattern is compatible with that of genes clustered in the *gsdf* locus of *O. latipes*, which are also expressed in previtellogenic oocytes (Gautier et al., 2011a). Thus, *gsdf* may participate in oogenesis in *P. olivaceus*. In the testis of *C. semilaevis*, *gsdf* is additionally expressed in spermatogonia and spermatids, suggesting *gsdf* function during spermatogenesis (Zhu et al., 2018). Both *C. semilaevis* and *P. olivaceus* are flatfish (Pleuronectiformes) (Figure 4). The additional expression in germ cells can be a feature either preserved or acquired during evolution. It will be interesting to know whether *gsdf* is also expressed in germ cells in other Pleuronectiformes.

In females, *gsdf* transcripts are present in granulosa cells. However, these granulosa cells can surround germs cells at early or late ovarian developmental stages depending on the species (Figure 4). The *gsdf* transcript is expressed at all follicular stages of *Gadus morhua* (Nagasawa et al., 2014), in vitellogenic follicles of *S. salar* (Kleppe et al., 2020), in previtellogenic follicles of *Oncorhynchus kisutch* (Luckenbach et al., 2008), and surround oogonia of *Scatophagus argus* (Jiang et al., 2019). The diverse expression in females suggests that Gsdf may have roles in early oogenesis, folliculogenesis and follicle maturation; and the regulatory elements controlling *gsdf* expression may be differentially acquired in different species.

The *gsdf* transcripts have been detected in undifferentiated XY gonad during the critical sex-differentiating period in juvenile *S. salar* (Lubieniecki et al., 2015), *O. niloticus* (Kaneko et al., 2015), *O. latipes* (Shibata et al., 2010), *Oryzias dancena* (Takehana et al., 2014), and *Oryzias sakaizumii* (Horie et al., 2016). Moreover, *gsdf* expression is correlated with the expression of the sex-determining genes, *dmy* in *O. latipes* and *O. sakaizumii*, *sox3* in *O. dancena*, and *sdY* in *S. salar* (Shibata et al., 2010; Takehana et al., 2014; Lubieniecki et al., 2015; Horie et al., 2016). This implies a role of *gsdf* in male sex differentiation downstream from initial sex determination.

In hermaphroditic species, expression of *gsdf* is also correlated with testicular development (Table 2). In protogynous teleosts, *H. trimaculatus*, *Epinephelus akaara*, and *M. albus*, *gsdf* is highly enriched during the transition from the ovarian phase to the testicular phase (Horiguchi et al., 2013; Chen et al., 2016; Zhu et al., 2016). This implies that *gsdf* may be involved in the transition into the male fate. In *H. trimaculatus*, the expression is in supporting cells surrounding gonial cell at early transition stage and in Sertoli cells surrounding spermatogonia at later transition stage, suggesting that *gsdf* might be involved in self-renewal and differentiation of spermatogonia during sex change (Horiguchi et al., 2013). In a protandrous teleost, *Acanthopagrus latus*, *gsdf* is highly expressed in the testicular zone of the ovo-testis, especially at the spermatogonia-dominant stage during spermatogenesis. This suggest that *gsdf* might be involved in the proliferation and differentiation of spermatogonia (Chen Y. et al., 2015).

**TABLE 2:** Expression of *gsdf* in hermaphroditic fishes.

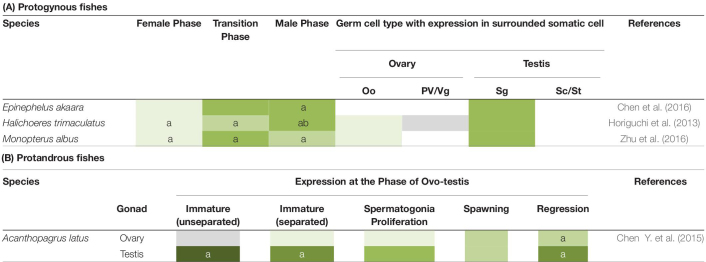

*^*a*^Expression in granulose cell (ovary) or Sertoli cell (testis). ^*b*^Expression in efferent duct. Oo, oogonia; PV, pre-vitellogenin; Vg, vitellogenin; Sg, spermatogonia; Sc, spermatocyte; St, spermatid. Color in each box indicates the expression level. White, data not available. Gray, no expression. Lighter green to darker green, lower expression to higher expression. Yellow, expression level is not specified in references.*

In non-teleost species, *C. orientalis*, *P. annectens*, and *L. menadoensis*, *gsdf* mRNA is enriched in the male gonad similar to that in teleosts (Forconi et al., 2013; Biscotti et al., 2018; Biscotti et al., 2020). This implies that *gsdf* may retain common functions in testicular development of different species.

## Function of *Gsdf*

The restricted but variable expression of *gsdf* in gonadal cells reflects its diverse roles during spermatogenesis and oogenesis (Figure 4). It can promote germ cell proliferation in some fish species, while inhibit proliferation in other fish species. It activates testicular development in many teleost species, and is a sex-determining gene for *O. luzonensis*.

### Modulation of Cell Proliferation

The *gsdf* transcript is present in Sertoli cells surrounding spermatogonia of several fish species (Table 1). It is an early gonadal marker when germ cells proliferate in European bass, *Dicentrarchus labrax*. However, *gsdf* transcript is reduced in germ cells of some males that enter meiosis precociously (Crespo et al., 2013). Knockdown of *gsdf* in the rainbow trout *O. mykiss* results in a decrease of primordial germ cells in larvae (Sawatari et al., 2007), indicating that *gsdf* might be important for the proliferation of germ cells especially spermatogonia. Proliferation assays further demonstrate this hypothesis. Recombinant Gsdf increases the number of BrdU-positive spermatogonia in a dose-dependent manner *in vitro* (Sawatari et al., 2007). BrdU staining further shows that most of the BrdU-positive cells are surrounded by Gsdf-positive supporting cells during female-to-male sex change of *H. trimaculatus* (Horiguchi et al., 2013). These reports all show that Gsdf is essential for the proliferation of spermatogonia for those fish during testicular development.

In contrast to a positive role in cell proliferation, Gsdf modulates cell division and prevents hyperplasia in some other species. Gsdf can prevent hyperproliferation of germ cells in *O. latipes* testis (Zhang et al., 2021). In *gsdf*-deficient juvenile ovaries, cystic germ cells undergo abnormal type-II division (Wu et al., 2019). These results suggest that Gsdf may modulate mitotic cell division. In *gsdf* mutant larvae of *O. latipes*, germ cell number in XY gonads is increased similar to that in XX gonad (Imai et al., 2015). Excessive number of germ cells is also present in adult testis. The mutant testis of *D. rerio* is fertile but hyperplastic, indicating the importance of *gsdf* in the regulation of testis size (Yan et al., 2017). Thus, Gsdf might play contradictory roles by promoting or curtailing germ cell proliferation in different species.

### Male Sex Determination

The sex of medaka is determined by a master sex-determining gene on the Y chromosome (Myosho et al., 2015). The most well-known fish sex-determining gene is *dmy* first found in Japanese medaka, *O. latipes* (Matsuda et al., 2002). In Philippine medaka, *O. luzonensis*, in which *dmy* is absent, *gsdfY* on the Y chromosome replaces *dmy* to act as a master gene for sex determination (Myosho et al., 2012). XX transgenic fish with overexpression of *gsdfY* develop into males (Myosho et al., 2012). This *gsdfY* is also functional in medaka species with other master sex-determining genes. For *O. latipes* and *O. dancena*, in which sex is determined by *dmy* and *sox3*, respectively, transgenic overexpression of *O. luzonensis gsdfY* leads to male development in most of XX fish (Myosho et al., 2012; Takehana et al., 2014). This indicates that downstream from GsdfY, Dmy, and Sox3, the steps involved in sex-specific gonad differentiation are probably similar in different medaka species. The promoter sequences of *gsdfY* in *O. luzonensis* differ from that of autosomal *O. latipes gsdf* in nine places. The *gsdfY* sequences at these sites are required for gene activation in a promoter reporter assay (Myosho et al., 2012). Thus, the unique proximal promoter sequence of *gsdfY* contributes to its predominant expression in the XY gonad. In sablefish, *Anoplopoma fimbria*, the *gsdf* locus is located downstream from sex-specific region, and is expressed in the XY gonad during the larval stage. Thus, *gsdf* may be the master sex-determining gene in *A. fimbria* (Rondeau et al., 2013).

### Testicular Development

The *gsdf* transcript is predominantly expressed in testis over ovary in most fish species. EE2 treatment of *O. latipes* causes male-to-female sex reversal, and expression of *gsdf* in XY gonad is decreased (Shibata et al., 2010). On the contrary, *gsdf* is highly expressed in male-like XX gonads in *O. latipes* with *cyp19a1* mutation or in *O. niloticus* with *foxl2* mutation (Zhang et al., 2017; Nakamoto et al., 2018). In addition, *gsdf* expression increases when ovarian follicles start to degenerate (Nakamoto et al., 2018). All these data imply that Gsdf has a role in testicular development.

All gain-of-function and loss-of-function studies show that Gsdf triggers testicular formation. Knocking down *gsdf* in a *C. semilaevis* testicular cell line, CSGC, leads to expression of female-related genes, *wnt4a*, *foxl2*, *star*, and *cyp19a1a* (Zhu et al., 2018). Knocking down *gsdf* in XY *O. niloticus* leads to ovarian differentiation (Kaneko et al., 2015). In knockout studies, all *gsdf* mutants of *O. latipes* and *O. niloticus* develop into females despite their genetic sex. Their secondary sex characteristics are also female. Their ovaries express female marker genes such as *foxl2* and *cyp19a1a*, and downregulates male marker genes, *dmrt1* and *cyp11b2* (Jiang et al., 2016; Zhang et al., 2016).

Male and female germ cells of *O. latipes* undergo different types of cell division. In XY larvae, germ cells carry out only intermittent type I division, in which only one or two germ cell is present in one cyst. In XX larvae, germ cells undergo both type I and continuous type II division forming clusters of germ cells (Saito et al., 2007). The *gsdf* mutant larvae contain more germ cells and their germ cell division is more female-type, indicating the induction of female development (Imai et al., 2015; Zhang et al., 2016). Besides, overexpression of *gsdf* in *O. niloticus* and *O. latipes* leads to testis morphology and male secondary sex characteristics. This indicates that *gsdf* is sufficient to initiate testicular differentiation (Kaneko et al., 2015; Zhang et al., 2016).

### Ovarian Development

Although *gsdf* is mainly expressed in the testis, the weak expression in the ovary suggests its other role in ovarian development. When *gsdf* is mutated, the ovaries of *D. rerio* and *O. latipes* become hyperplastic with most follicles arrested in the primary growth and previtellogenic stage (Guan et al., 2017; Yan et al., 2017). In *O. latipes*, *gsdf* mutation leads to restrained oocyte growth, and ovarian maturation is compromised (Guan et al., 2017). In *gsdf*-deficient juvenile females, cystic germ cells undergo abnormal type-II division, causing ovarian hyperplasia (Wu et al., 2019).

In *D. rerio*, ovarian defects of *gsdf* mutants include a decrease of estrogen production, downregulation of genes for steroid biosynthesis, and decreased estrogen action. Furthermore, granulosa marker genes including *cyp19a1a* and *gata4* are downregulated in mutants (Yan et al., 2017). Therefore, *gsdf* is essential for the maturation or maintenance of granulosa cells, which are essential for the secretion of estrogen and the ensuing vitellogenin synthesis (Yan et al., 2017).

### Regulation of *Gsdf*

Although the mechanism of *gsdf* regulation is not fully understood, there are some hints. Some of the regulatory pathway is common, while others are distinct among species (Figure 4). In Ovalentaria species, *gsdf* can act downstream from and be regulated by the master sex-determination genes. The sex-determining gene on the Y chromosome of *O. dancena* is *sox3*. The *gsdf* transcript is upregulated in XX gonad by *sox3* overexpression while downregulated in the gonad of XY *sox3* mutants (Takehana et al., 2014). In *O. latipes*, the *gsdf* promoter contains two putative binding sites for the sex-determining protein, Dmy (Zhang et al., 2016). ChIP and luciferase assay show that Dmy can directly bind to the *gsdf* promoter and enhance its activity in a dose-dependent manner (Chakraborty et al., 2016). It is interesting to investigate whether in other Ovalentaria species, Gsdf also act as the downstream factor of master sex-determining genes.

The regulation of *gsdf* among different species shares some common elements. Putative binding elements were found. In mammals, Sf1 and Dmrt1 are required for early testicular development after the initiation of sex determination (She and Yang, 2017). The proximal promoter of *gsdf* in *S. argus* and *O. niloticus* genomes also contain binding sites for Sf1 and Dmrt1 required for the activation of *gsdf* promoter (Jiang et al., 2016; Jiang et al., 2019). *Orechromis niloticus* and *S. argus* are species from two evolutionarily distant clades among Percomorphaceae (Figure 4). This implies that these *gsdf* regulators are conserved among these fish species during evolution.

In *D. rerio*, six DNA binding motifs, E-box, SOX, GATA, SF1, CEBP/AP2/IL6RE, and one unknown motif, were found within 2-kb proximal promoter of *gsdf*, which are conserved among three other teleosts, *Gasterosteus aculeatus*, *Takifugu rubripe*s, and *O. latipes*. The transcription factors of most of the motifs are expressed in Sertoli cells or granulosa cells (Gautier et al., 2011b), but their functions in regulating *gsdf* expression has not been shown. The functions of *gsdf* are diverse among different species. Thus, each species might acquire its own regulatory motif resulting in distinct regulation of *gsdf*.

## Receptor of Gsdf

Although *gsdf* has been found widely in many species, the identity of Gsdf receptor still remains illusive. Being a Tgfβ family member, Gsdf may bind to known Tgfβ receptors. Thus, studies of other Tgfβ proteins, including Bmp and Amh, and their receptor can provide some clues. In *O. latipes*, mutation of *amhrII* results in gonadal hyperplasia and male-to-female sex reversal in some XY fish, similar to *gsdf* mutant phenotypes (Morinaga et al., 2007; Imai et al., 2015; Zhang et al., 2016). In *D. rerio*, mutations of genes encoding *amh*, *bmpr1bb*, and *bmpr2a* all lead to gonadal hyperplasia and accumulation of immature oocytes, mimicking the phenotypes of *gsdf* mutant (Neumann et al., 2011; Yan et al., 2017, 2019; Zhang Z. et al., 2020). Thus, Gsdf might share the same receptors with other Tgfβ proteins. However, these mutants have additional phenotypes including female-biased sex ratio in *amh* mutant and the accumulation of immature spermatogenic cells in testis of *bmpr1bb* mutant (Neumann et al., 2011; Yan et al., 2019). This also raises the possibility that Gsdf may bind to its own yet unidentified receptor.

## Conclusion

Gonadal somatic cell-derived factor is a factor that exists mostly in teleosts and functions mainly in gonad differentiation. Its expression, regulation and function, however, change substantially in different species. In this review, we summarize the existence, expression, regulation and function of *gsdf*, comparing their commonality and diversity during species evolution. The presence of *gsdf* contributes to the difference of gonad development between teleosts and most tetrapods, while its varied upstream and downstream regulation also results in diversity among teleost species.

Gonadal somatic cell-derived factor is present in both male and female gonads. In males, the expression and regulation of *gsdf* can be common or species-specific. The *gsdf* gene is usually expressed in Sertoli cells surrounding spermatogonia, suggesting its involvement in self-renewal, proliferation and differentiation of spermatogonia. Moreover, *gsdf* is additionally expressed in efferent ducts in *H. trimaculatus* and species belonging to Ovalentaria (Figure 4). In species from two distant clades among Percomorphaceae, *gsdf* is activated by Sf1 and Dmrt1, implying the conserved common regulation (Figure 4). In Ovalentaria, Gsdf functions in male sex determination, either acting as a sex-determining gene or working downstream from sex-determining factors (Figure 4). Thus, the species-specific regulation of *gsdf* is preserved in limited clades during evolution.

In females, *gsdf* is less expressed and mostly in granulose cell surrounding different types of germ cell in different teleost species. Besides participating in testicular development, Gsdf plays a major role in ovarian development in *D. rerio* (Yan et al., 2017). The distinct ovarian function in *D. rerio* and the diverse expression suggest that the regulation of *gsdf* is acquired differently during evolution.

Gonadal somatic cell-derived factor is a gonadal protein of multiple functions widely expressed in many species. The mechanism by which Gsdf functions in different species and developmental stages remains an interesting question. Furthermore, little is known about the upstream and downstream regulation of Gsdf. The identification of Gsdf receptor and the analysis of *gsdf* promoters in different species will be the key. With this knowledge at hand, one can then better understand the multiple functions of Gsdf as a result of its diverse regulation.

## Author Contributions

C-WH and B-CC conceived the ideas, organized the literature, analyzed the data, and wrote the manuscript. B-CC conceived the ideas, analyzed the data, and wrote the manuscript.

## Conflict of Interest

The authors declare that the research was conducted in the absence of any commercial or financial relationships that could be construed as a potential conflict of interest.
